# Natural podophyllotoxin analog 4DPG attenuates EMT and colorectal cancer progression via activation of checkpoint kinase 2

**DOI:** 10.1038/s41420-021-00405-3

**Published:** 2021-01-26

**Authors:** Archana Katoch, Debasis Nayak, Mir Mohd. Faheem, Aviral Kumar, Promod Kumar Sahu, Ajai Prakash Gupta, Lekha Dinesh Kumar, Anindya Goswami

**Affiliations:** 1 Academy of Scientific & Innovative Research (AcSIR), Ghaziabad, 201002 India; 2 Cancer Pharmacology Division, CSIR-Indian Institute of Integrative Medicine, Canal Road, Jammu, Jammu and Kashmir 180001 India; 3 Division of Pharmaceutics and Pharmacology, College of Pharmacy, The Ohio State University, Columbus, OH 43210 USA; 4 School of Biotechnology, University of Jammu, Jammu, Jammu and Kashmir 180006 India; 5 Cancer Biology, CSIR-Centre for Cellular & Molecular Biology, Hyderabad, Telangana 500007 India; 6 Quality Control and Quality Assurance Division, CSIR-Indian Institute of Integrative Medicine, Jammu, Jammu and Kashmir 180001 India

**Keywords:** Cancer therapeutic resistance, Metastasis

## Abstract

Epithelial–mesenchymal transition (EMT) is critical for the metastatic dissemination of cancer cells and contributes to drug resistance. In this study, we observed that epithelial colorectal cancer (CRC) cells transiently exposed to 5-fluorouracil (5-FU) (a chemotherapeutic drug for CRC) as well as 5-FU-resistant cells (5-FU-R) develop EMT characters as evidenced by activation of Vimentin and augmented invasive properties. On the other hand, 4DPG (4′-demethyl-deoxypodophyllotoxin glucoside), a natural podophyllotoxin analog attenuates EMT and invadopodia formation abilities of HCT-116/5-FU-R and SW-620/5-FU-R cells. Treatment with 4DPG restrains Vimentin phosphorylation (Ser38) in 5-FU-R cells, along with downregulation of mesenchymal markers Twist1 and MMP-2 while augmenting the expression of epithelial markers E-cadherin and TIMP-1. Moreover, 4DPG boosts the tumor-suppressor protein, checkpoint kinase 2 (Chk2) via phosphorylation at Thr68 in a dose-dependent manner in 5-FU-R cells. Mechanistically, SiRNA-mediated silencing of Chk2, as well as treatment with Chk2-specific small-molecule inhibitor (PV1019), divulges that 4DPG represses Vimentin activation in a Chk2-dependent manner. Furthermore, immunoprecipitation analysis unveiled that 4DPG prevents complex formation between Vimentin and p53 resulting in the rescue of p53 and its nuclear localization in aggressive 5-FU-R cells. In addition, 4DPG confers suitable pharmacokinetic properties and strongly abrogates tumor growth, polyps formation, and lung metastasis in an orthotopic rat colorectal carcinoma model. In conclusion, our findings demonstrate 4DPG as a targeted antitumor/anti-metastatic pharmacological lead compound to circumvent EMT-associated drug resistance and suggest its clinical benefits for the treatment of aggressive cancers.

## Introduction

Colorectal cancer (CRC) is one of the most common malignant diseases in developed countries, and it remains a major public health problem^1,2^. Survival rates of patients with CRC have improved in the past few years, probably due to earlier diagnosis and improved treatment of this disease. In spite of this, following potentially curative surgery, ~30% of patients eventually develop local tumor recurrence or distant metastasis, resulting in an overall poor prognosis^3,4^. Such patients usually receive chemotherapy in combination with targeted monoclonal antibody therapy yielding response rates of, at best, 50%^3,5^. 5-Fluorouracil (5-FU) is a pyrimidine analog chemotherapeutic drug currently being used in clinical practice since the 1950s as a first-line treatment option for CRC^6,7^. Although the drug efficiently reduces primary tumor mass initially, cases of recurrence have been reported frequently indicating the presence of chemoresistant cancer cells^7^. Several mechanisms have been identified in recent years through which cancer cells escape chemotherapy-induced cell death. These include the development of stemness, entry into quiescence, and the onset of invasion and metastasis; the latest being the foremost reason for maximum patient mortality^8–10^. Therefore, understanding the molecular mechanisms governing invasion–metastasis cascade and drug resistance could be fruitful in designing better-targeted therapies against CRC.

The invasion–metastasis process consists of a series of highly synchronized events including local invasion, intravasation into the adjacent blood vessels, extravasation at a distant organ site, and formation of secondary/metastatic tumors^11^. Recent research has demonstrated that the epithelial–mesenchymal transition (EMT), a key developmental program, also initiates and facilitates the invasion–metastasis process in cancer^12,13^. A wide array of molecular alterations accompany acquisition of EMT along with phenotypic changes such as elongated cellular morphology, cell scattering, and migration characteristics. In particular, high expression of pro-EMT transcription factors/mesenchymal markers such as N-cadherin, Twist1, ZEB-1, Vimentin, etc., and diminished expression of epithelial markers/cell adhesion molecules E-cadherin, Occludin, cytokeratin, and ZO-1 are frequently observed in many cancers undergoing EMT^14^. Moreover, a growing body of evidence also establishes profound contributions of EMT towards chemotherapeutic resistance (gemcitabine, 5-FU, cisplatin, etc.) in many cancers including CRC and pancreatic carcinoma^15–18^. Furthermore, in CRC, tumor cells undergoing EMT are histologically represented by the presence of tumor buds defined as single cells or small clusters of dedifferentiated tumor cells at the invasive front^19,20^. Tumor budding is a predictor of lymph node metastasis, vascular and lymphatic invasion, distant metastasis, local recurrence, and poor disease-specific survival duration^21,22^. Moreover, the Union for International Cancer Control (UICC) identifies tumor budding as a critical and additional prognostic factor^23^. Therefore, it is very likely that further research into the molecular as well as genomic targets and biomarkers underpinning EMT regulation holds tremendous potential in identifying the subset of patients who are at the highest risk of developing metastasis in CRC.

Checkpoint kinase 2 (Chk2) is a key signal transducer protein of the DNA damage checkpoint pathway, which is activated by ataxia–telangiectasia-mutated (ATM) protein kinase in response to DNA double-strand breaks. ATM phosphorylates Chk2 at Thr68, which upon activation regulates the functions of various downstream substrates including p53, CDC25, and BRCA1^24–26^. Chk2 acts as a key tumor-suppressor protein that provides a barrier to tumorigenesis due to DNA damage by halting the cell cycle and taking decisions on whether to proceed for DNA-damage repair/apoptosis or senescence, depending on the severity of the DNA damage^27–30^. Recently, we substantially addressed a novel anti-metastatic role of Chk2 in preventing Twist1-mediated EMT and invasion of p53-defective aggressive cancer cells^31^. In this respect, our research group has reported 4DPG, a small-molecule anticancer agent (isolated from the medicinal plant *Podophyllum hexandrum*) that modulates Chk2 in malignant cells^32^. In the present study, we demonstrate a mechanistic basis of the potential of 4DPG in abrogating EMT and invasion of drug-resistant CRC cells by modulating Vimentin expression. In addition, we also study the pharmacokinetics and in vivo efficacies of 4DPG against colorectal tumor growth and metastases.

## Results

### Elevated Vimentin expression and invasive phenotypes are common following transient treatment of 5-FU as well as in 5-FU-resistant colorectal cancer cells

**Fig. 1:**
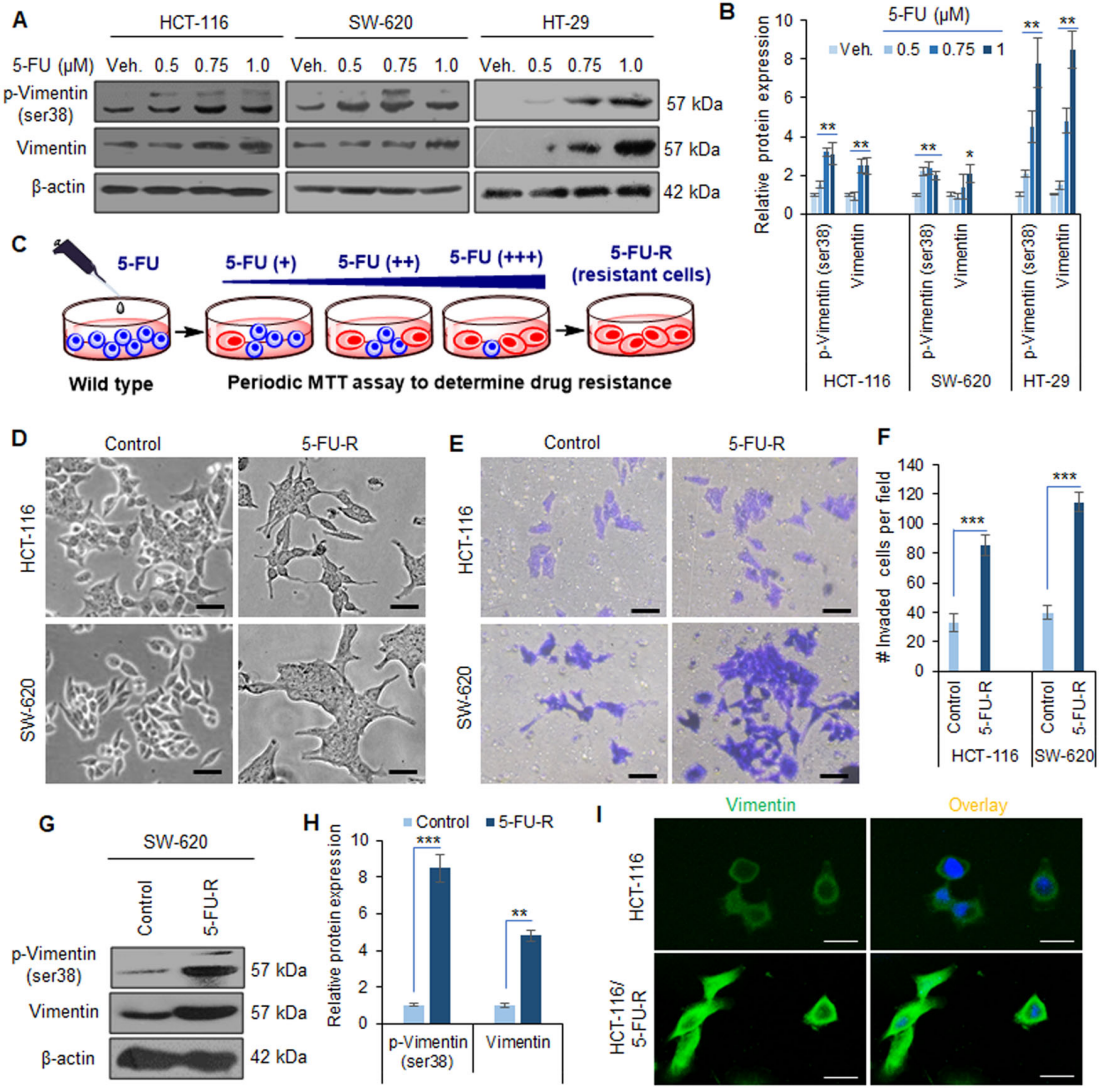
Vimentin activation and the onset of EMT in CRC cells transiently exposed to 5-FU, as well as in 5-FU-resistant population. **A** Western blotting analysis depicting protein expression of p-Vimentin (Ser38) and Vimentin in HCT-116, SW-620, and HT-29 cells treated with indicated concentrations of 5-FU for 24 h. β-Actin expression was considered as an endogenous loading control. **B** Densitometry analysis of western blot bands presented above showing relative protein expression at each concentration of 5-FU. *n* = 3, error bars: mean ± SD; *P < 0.05, **P < 0.01. **C** Model showing the procedure followed for generating the 5-FU-R cells from the wild-type CRC cells (details in “Materials and methods”). **D** Phase contrast images showing the morphology of wild-type HCT-116 and SW-620 cells (control) versus 5-FU-resistant (HCT-116/5-FU-R, SW-620/5-FU-R) cells. Scale bar: 50 µm. **E** Matrigel invasion assay results showing the differences in invasion abilities of wild-type (control) versus 5-FU-R CRC cells. Scale bar: 50 µm. **F** Quantification of the number of invaded cells per field from the above invasion assay experiment. *n* = 3, error bars: mean ± SD; ***P < 0.001. **G** Western blots depicting protein expression of p-Vimentin (Ser38) and Vimentin in wild-type (control) versus SW-620/5-FU-R cells. β-Actin expression was considered as an endogenous loading control. **H** Densitometry analysis showing the relative protein expression in each band of western blots presented above. *n* = 3, error bars: mean ± SD; **P < 0.01, ***P < 0.001. **I** Immunocytochemistry results representing the Vimentin expression in wild-type (HCT-116) versus HCT-116/5-FU-R cells. Scale bars: 50 µm.

Vimentin belongs to the family of intermediate filament proteins that are known to function in maintaining the shape as well as the integrity of the normal mesenchymal cells^33,34^. However, it has been established to regulate the cell shape and migration capability during EMT, a phenomenon critical to metastasis of cancer cells^35,36^. To investigate any plausible contribution of Vimentin to 5-FU resistance in CRC, we treated a panel of three epithelial colorectal carcinoma cell lines (HCT-116, SW-620, and HT-29) with increasing concentrations of the said drug, transiently (24 h). Western blots were generated for the whole-cell lysates obtained from the above set of experiments. Since the phosphorylated Vimentin is an important signal transducer during the EMT process, we analyzed p-Vimentin (ser38) as well as total Vimentin levels in these lysates. A substantial boost in the p-Vimentin (2–7.8-fold) concomitant with increasing Vimentin expression levels (2.1–8.5-fold) was observed at the sub-toxic concentration of 5-FU (1 µM) in these cells (Fig. 1A, B).

We then attempted to investigate the phenotypical and biochemical alterations associated with chronic exposure of 5-FU to CRC cells. Accordingly, 5-FU-resistant (5-FU-R) cells were prepared through a rigorous selection strategy in presence of increasing concentrations of the drug for a prolonged period (at least 20 weeks) (Fig. 1C). The resulting HCT-116/5-FU-R, SW-620/5-FU-R, and HT-29/5-FU-R cells showed acquired resistance up to 50, 60, and 45 µM of 5-FU, respectively. The microscopic observation of the HCT-116/5-FU-R and SW-620/5-FU-R cells revealed the development of mesenchymal phenotypes with remarkable changes such as elongated cell shape and scattered colony morphology, which was clearly noticeable and distinct compared to the respective parental cells (Fig. 1D). Next, we wanted to assess if the aggressive drug-resistant phenotypes also influenced the invasion ability of these cancer cells. The Boyden chamber matrigel invasion assay results depicted a significant increase in the number of invaded cells per field: 2.55-fold and 2.89-fold in HCT-116/5-FU-R and SW-620/5-FU-R cells, respectively, compared to the parental cells (Fig. 1E, F). Since Vimentin expression was augmented upon transient exposure of 5-FU to CRC cells, we were curious to examine whether the expression of thispro-EMT marker upholds invasive phenotypes in drug-resistant cells. The western blotting analysis results unveiled a sharp increase in p-Vimentin (ser38) levels (8.5- and 1.85-fold) along with augmentation of total Vimentin (4.8- and 2.45-fold) in SW-620/5-FU-R and HT-29/5-FU-R cells compared to the control cells (Fig. 1G, H, Supplementary Fig. S1A, B). The immunocytochemistry results further corroborated higher Vimentin expression levels in drug-resistant cells compared to the respective parental cells (Fig. 1I and Supplementary Fig. S1C). Together, these findings demonstrate that the exposure to chemotherapeutic agents (5-FU) promotes Vimentin activation and the development of EMT phenotypes in CRC.

### 4DPG triggers Chk2 expression, suppresses Vimentin activation, and EMT in 5-FU-resistant CRC cells

**Fig. 2:**
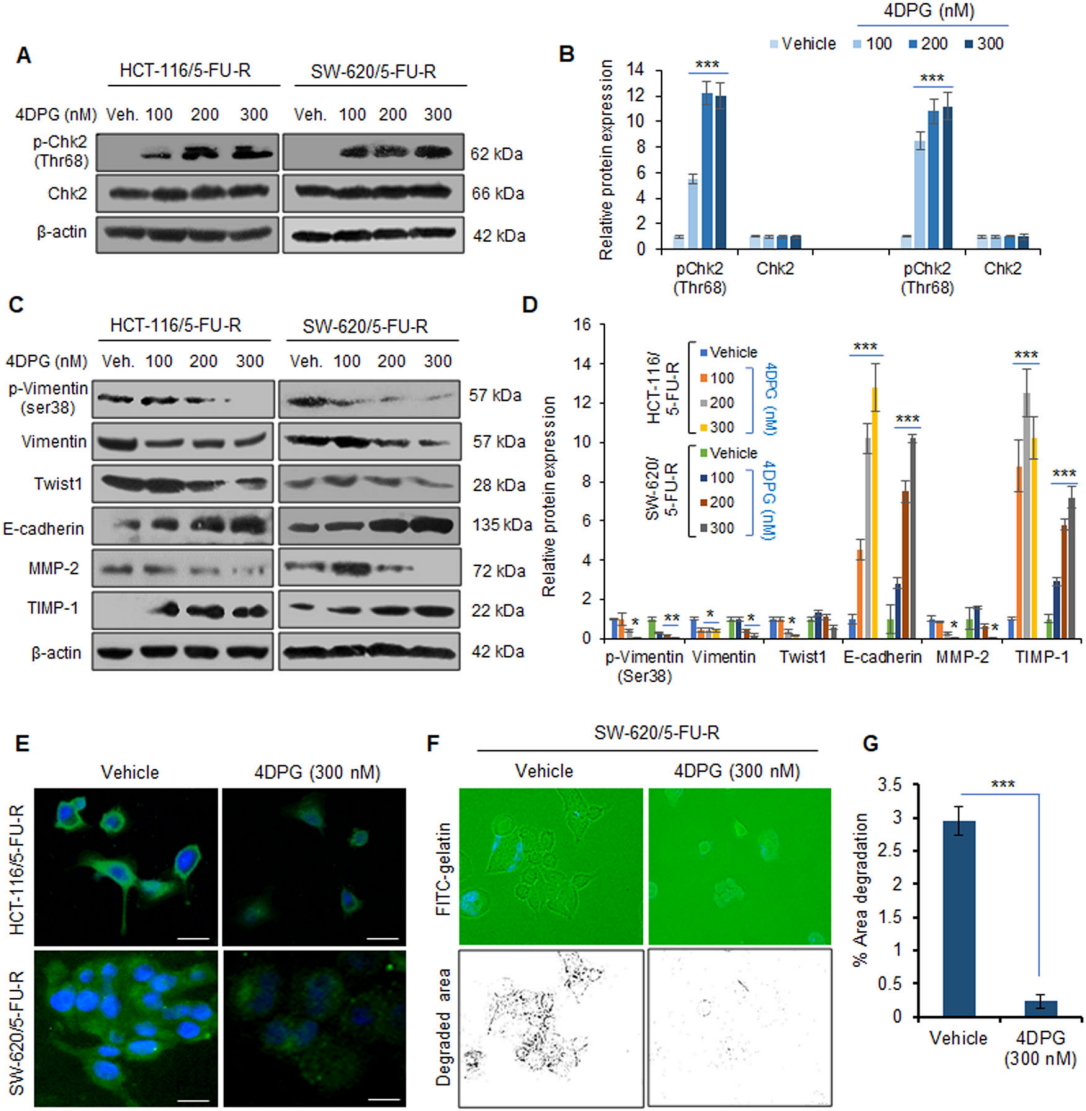
4DPG activates Chk2 and attenuates EMT in 5-FU-R CRC cells. **A** Western blotting analysis results portraying protein expression of p-Chk2 (Thr68) and Chk2 in HCT-116/5-FU-R and SW-620/5-FU-R cells in response to increasing concentrations of 4DPG, treated for 48 h. β-Actin expression was considered as endogenous loading control. **B** Densitometry analysis showing relative protein expression of western blot bands presented above. *n* = 3, error bars: mean ± SD; ***P < 0.001. **C** Western blots depicting protein expression of EMT markers in HCT-116/5-FU-R and SW-620/5-FU-R cells in response to increasing concentrations of 4DPG, treated for 48 h. β-Actin expression was considered as endogenous loading control. **D** Densitometry analysis of western blots presented in (**C**) showing quantification of protein expression. *n* = 2, error bars: mean ± SD; *P < 0.05, **P < 0.01, ***P < 0.001. **E** Immunocytochemistry results displaying the protein expression levels of Vimentin in HCT-116/5-FU-R and SW-620/5-FU-R cells, treated with vehicle and/or 4DPG (300 nM) for 48 h. Scale bars: 50 µm. **F** In situ fluorescent gelatin degradation assay results showing the invadopodia/footprints of invaded SW-620/5-FU-R cells treated with vehicle and/or 4DPG at the indicated concentration for 48 h. Blue stains over the green background display DAPI staining of nuclei of cells. **G** Quantification of the area of degradation through Image J software. *n* = 3, error bars, mean ± SD; ***P < 0.001.

4DPG, a podophyllotoxin class of natural product anticancer compound (Supplementary Fig. S2A) has been recently described by our research group for its potent antitumor and anti-metastatic properties against aggressive cancers^31,32^. Further, 4DPG augments phosphorylation and activation of Chk2 (Thr68) leading to suppression of EMT and invasiveness of p53-defective cancer cells^31^. To investigate the effect of 4DPG on phosphorylation and expression levels of Chk2 in HCT-116/5-FU-R, SW-620/5-FU-R, and HT-29/5-FU-R cells, we treated these cells for 48 h with increasing concentrations of the compound, and whole-cell lysates were subjected to western blotting analysis. Robust increase in p-Chk2 (Thr68) levels was observed (12-fold in HCT-116/5-FU-R, 11.2-fold in SW-620/5-FU-R, and 7.9-fold in HT-29/5-FU-R) at the sub-toxic concentration (300 nM) of the compound, whereas, the expression of Chk2 did not alter significantly in 4DPG-treated cells compared to the vehicle (Fig. 2A, B, Supplementary Fig. S2B, C). Since our previous report has demonstrated that Chk2 activation negatively regulates EMT, coherently, we examined whether the expression of the mesenchymal/epithelial markers could be modulated by 4DPG in these 5-FU-R cells. Interestingly, the immunoblot data revealed remarkable downregulation of p-Vimentin (ser38), Vimentin, and associated pro-EMT markers such as Twist1, and MMP-2, while concurrent amplification in the epithelial markers, E-cadherin (upto 12.8-fold in HCT-116/5-FU-R and 10.2-fold in SW-620/5-FU-R) and TIMP-1 (up to 12.5-fold in HCT-116/5-FU-R and 7.2-fold in SW-620/5-FU-R) was detected in these cells in presence of 4DPG (Fig. 2C, D). The immunocytochemistry data also demonstrated a considerable decrease in Vimentin expression level in HCT-116/5-FU-R and SW-620/5-FU-R cells treated with 4DPG (300 nM) for 48 h (Fig. 2E). To determine the effect of 4DPG on the invasion abilities of these 5-FU-R cells, we performed in situ fluorescent gelatin degradation assay following our pre-standardized protocol^31,37^. In physiological conditions, this assay mimics the extracellular matrix degradation by aggressive tumor cells undergoing invasion–metastasis process. We observed sufficient invadopodia formation (footprints/degradation over the FITC-gelatin matrix) in SW-620/5-FU-R cells treated with vehicle, whereas invadopodia formation and the area of degradation over the gelatin matrix was significantly reduced in 4DPG-treated conditions indicating loss of invasive potential (Fig. 2F, G). Taken together, these results imply that 4DPG stimulates Chk2, suppresses Vimentin activation, EMT, and invasion of 5-FU-R CRC cells.

### 4DPG modulates p53 expression by repressing Vimentin in a Chk2-dependent manner

**Fig. 3:**
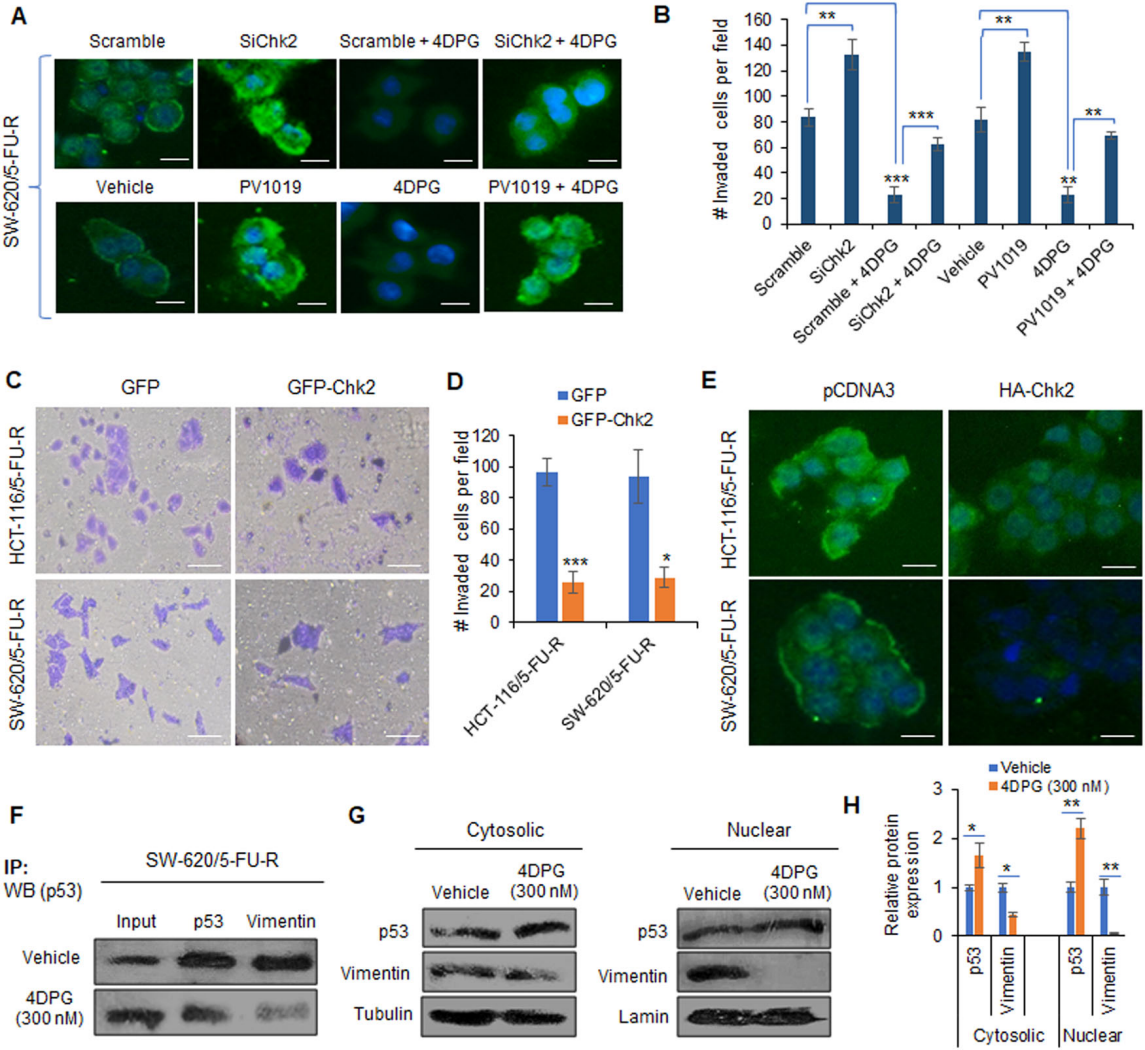
Chk2-dependent role of 4DPG in modulating p53 and repressing Vimentin expression in 5-FU-R cells. **A** Immunocytochemistry results showing the Vimentin expression level in SW-620/5-FU-R cells treated/transfected with scramble, SiChk2, scramble plus 4DPG, SiChk2 plus 4DPG, vehicle, PV1019, 4DPG, and PV1019 plus 4DPG for 48 h. Scale bars: 50 µm. **B** Quantification of number of invaded HCT-116/5-FU-R cells exposed to the above treatment conditions for 48 h. *n* = 2, error bars, mean ± SD; **P < 0.01, ***P < 0.001. **C** Matrigel invasion assay results showing invaded HCT-116/5-FU-R and SW-620/5-FU-R cells following 48 h of transient transfection with GFP and GFP-Chk2 plasmid construct. Scale bars: 50 µm. **D** Quantification of the number of invaded cells per field from the above experimental conditions. *n* = 3, error bars, mean ± SD; *P < 0.05, ***P < 0.001. **E** Immunocytochemistry results displaying Vimentin expression levels in HCT-116/5-FU-R and SW-620/5-FU-R cells after 48 h of transient transfection with pCDNA3 and/or HA-Chk2 plasmid construct. Scale bars: 50 µm. **F** Immunoprecipitation with Vimentin or p53 followed by western blotting analysis of p53 in SW-620/5-FU-R cells treated with vehicle and/or 4DPG (300 nM) for 48 h. **G** Cytosolic (left panel) and nuclear (right panel) extracts of SW-620/5-FU-R cells treated with vehicle and/or 4DPG (300 nM) for 48 h showing protein expression of Vimentin and p53. Tubulin and lamin expression was considered as endogenous loading controls for cytosolic and nuclear fractions, respectively. **H** Densitometry analysis of the above western blots showing relative protein expression of p53 and Vimentin in cytoplasmic and nuclear extracts. *n* = 3, error bars: mean ± SD; *P < 0.05, **P < 0.01.

To examine whether 4DPG-mediated repression of Vimentin and invasiveness is a Chk2-dependent process, we performed genetic silencing of endogenous Chk2 in the 5-FU-R cells using target-specific SiRNAs. Our immunocytochemistry data revealed remarkable augmentation of Vimentin expression in SiChk2-transfected SW-620/5-FU-R cells compared to the scramble transfection (Fig. 3A). On the contrary, a substantial reduction in Vimentin level was clearly visible in scramble plus 4DPG-treated cells. However, 4DPG was unable to exert its blocking effects in Chk2-silenced condition; only mild inhibition of Vimentin level was apparent in SiChk2 plus 4DPG-treated conditions (Fig. 3A). We also treated these 5-FU-R cells with Chk2-specific small-molecule inhibitor PV1019^38^, which showed increased Vimentin level similar to the case of SiChk2 transfection (Fig. 3A). Like the SiChk2 plus 4DPG condition, negligible inhibition of Vimentin level resulted in PV1019 plus 4DPG-treated conditions (Fig. 3A). Similarly, a remarkable increase in the number of invaded cells was achieved in SiChk2 as well as PV1019-treated conditions and the effect of 4DPG to block the invasion abilities of these cells was distinctly reduced in SiChk2 and PV1019 pre-treated conditions (Fig. 3B and Supplementary Fig. S3). These findings suggest the Chk2-dependent role of 4DPG in inhibiting Vimentin and abrogating invasion of drug-resistant cells.

We then poised to investigate the effect of ectopic overexpression of Chk2 on the aggressiveness of these 5-FU-R cells. Accordingly, SW-620/5-FU-R and HCT-116/5-FU-R cells were transiently transfected with GFP or GFP-Chk2 plasmid constructs and subjected to a Transwell invasion assay. Interestingly, corresponding to the influence of 4DPG, we noticed a significant decrease in the number of invaded cells per field: 3.8-fold and 3.27-fold reduction in GFP-Chk2 transfected HCT-116/5-FU-R and SW-620/5-FU-R cells, respectively, compared to the GFP-transfection (Fig. 3C, D). Furthermore, a considerable decline in Vimentin expression was clearly visible in both the GFP-Chk2-transfected 5-FU-R cells compared to the respective control (Fig. 3E). The data demonstrates that the Chk2 overexpression exerts effects similar to 4DPG treatment in regulating Vimentin expression and invasion of these malignant cells.

Moreover, to gain mechanistic insights into how 4DPG-induced activation of Chk2 suppresses EMT and invasiveness, we examined the downstream substrates of Chk2. Tumor suppressor p53 acts as a direct substrate of Chk2 that regulates cell cycle, DNA repair, or apoptosis of cancer cells in response to DNA damage^27,39^. We attempted to investigate whether there any molecular interaction takes place between Vimentin and p53 in these 5-FU-R cells. Our immunoprecipitation analysis unveiled that 4DPG potentially interrupted the association of Vimentin and p53 in SW-620/5-FU-R cells. Certainly, ample p53 protein immunoprecipitated with Vimentin in vehicle-treated cells, whereas negligible p53 expression was detected in Vimentin pull-down lysates from SW-620/5-FU-R cells treated with 4DPG for 48 h (Fig. 3F). Since 4DPG disrupted the Vimentin–p53 association, we were curious to see the consequences on p53 expression in various cellular compartments. We separated cytosolic and nuclear fractions of SW-620/5-FU-R cells treated with vehicle and/or 4DPG and conducted western blotting analysis considering relevant loading controls. In cytosolic fractions (left panel), it was observed 1.65-fold augmentation in p53 expression parallel to 2.2-fold downregulation of Vimentin levels in 4DPG (300 nM) treated 5-FU-R cells. More importantly, nuclear fractions (right panel) showed remarkable alterations in the expression of these proteins (2.2-fold increase in p53 and 20-fold decrease in Vimentin levels) in the 4DPG-treated conditions compared to the vehicle (Fig. 3G, H). These results demonstrate that 4DPG prevents the interaction between Vimentin and p53 and promotes cellular levels of p53 in these aggressive 5-FU-R cells.

### Assessment of solubility, distribution coefficient, hemocompatibility, and pharmacokinetic (PK) properties of 4DPG

Solubility and distribution coefficient are among the major physicochemical properties of a drug that greatly determine its absorption in physiological compartments^40^. 4DPG was found to be insoluble in phosphate buffered saline (PBS, pH 7.4), simulated intestinal fluid (SIF, pH 6.8), and simulated gastric fluid (SGF, pH 1.2) with solubility values 936.42 ± 117, 712.92 ± 172, and 539.38 ± 91 µg/mL, respectively. The distribution coefficient (logD, pH 7.4) of 4DPG was determined to be 0.815 ± 0.19, which indicates the slightly lipophilic nature of the molecule. For administration to animals, the appropriate quantity of 4DPG was formulated in the pre-calculated volume of saline with the help of a solubilizer (Tween-80). Next, to assess the hemocompatibility of 4DPG and establish if the compound could be considered safe for intravenous (i.v.) administration, we performed in vitro hemolysis assay by incubating the RBCs with increasing concentrations of the molecule. Interestingly, negligible hemolysis (5.8%) was detected at the highest tested concentration of the molecule (20 µg/mL), which was very much similar to the hemolysis observed in the case of positive control (PBS) demonstrating 4DPG is nontoxic and suitable for i.v. administration (Supplementary Fig. S4A, B).

Subsequently, to evaluate the exposure of 4DPG in healthy animals, we performed PK analysis in Balb/c mice by administering a single dose (5 mg/kg) of the compound intravenously. The initial concentration (C_0_) in plasma was found to be 10165.18 ng/mL with the half-life (*t*_1/2_): 0.653 h and area under the curve (AUC_0-∞_): 10104.05 ng*h/mL. The volume of distribution (Vd) and clearance (Cl) was determined to be 0.466 L/Kg and 0.495 L/h/kg, respectively, along with a mean residence time (MRT) of 0.306 h (Table 1 and Supplementary Fig. S4C). The obtained PK parameters of 4DPG indicate its adequate absorption and distribution in the body as well as rapid clearance demonstrating the compound as an appropriate candidate for further in vivo studies in diseased animal models.

**Table 1:** Pharmacokinetic parameters of 4DPG in Balb/c mice intravenously administered with a single dose of 5 mg/kg/body weight.

PK parameters	Values
C initial (C_0_) (ng/mL)	10165.18
Half-life (*t*_1/2_) (h)	0.653
Clearance (Cl) (L/h/kg)	0.495
Volume of distribution (Vd) (L/kg)	0.466
Area under curve (AUC_0-t_) (ng*h/mL)	10068.884
Area under curve (AUC_0-∞_) (ng*h/mL)	10104.053
Mean residence time (MRT) (h)	0.306
Time point considered in *t*_1/2_ calculation	0.5–1 h

### 4DPG inhibits tumor growth and lung metastasis in orthotopic rat colorectal carcinoma model

**Fig. 4:**
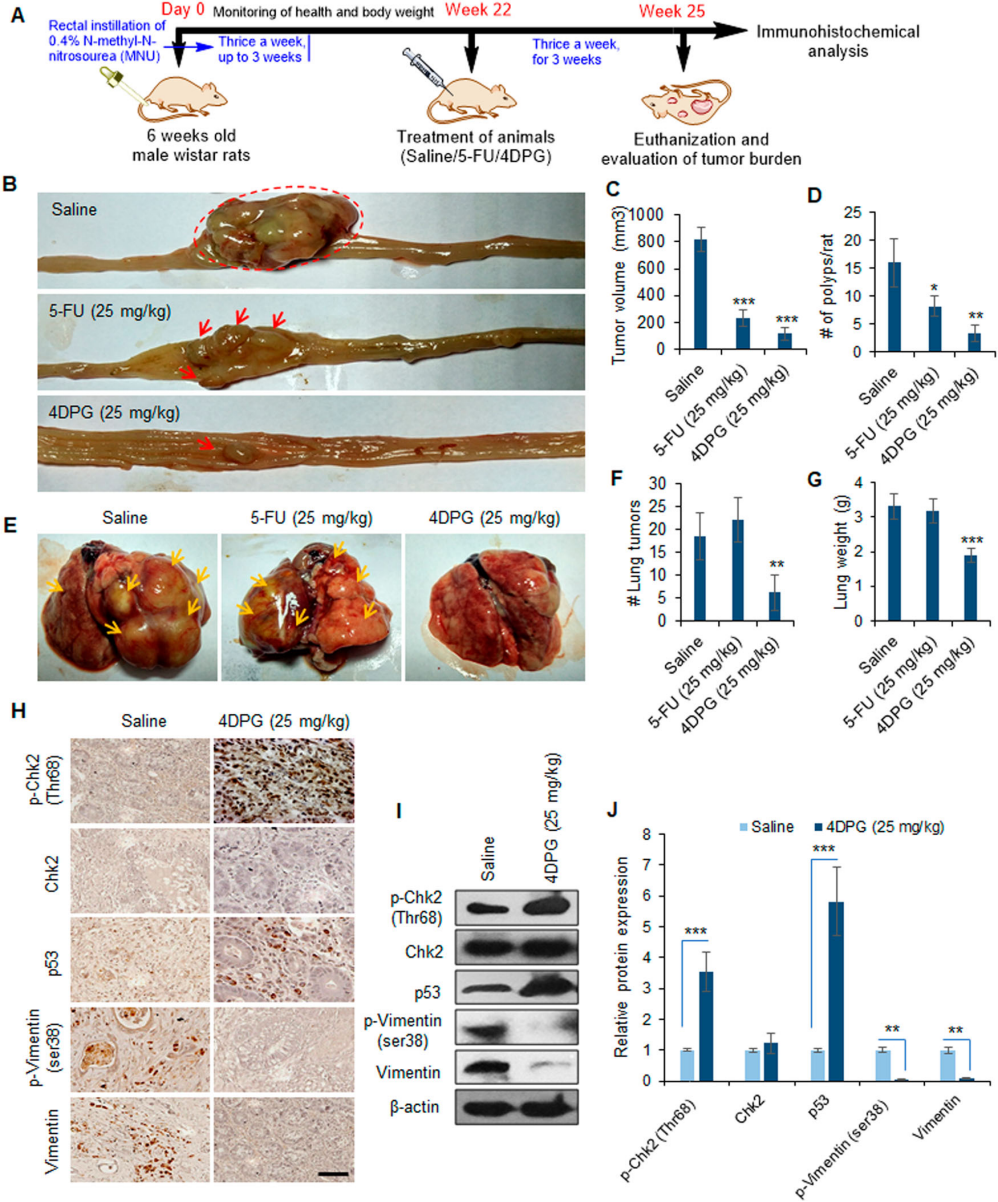
4DPG inhibits tumor growth and lung metastases in orthotopic rat CRC model. **A** Model depicting the procedure adopted for generating the carcinogen-induced orthotopic rat CRC model, treatment strategies of 4DPG, evaluation of tumor burden, and further biochemical analysis. **B** Representative images of large bowel showing the colorectal tumor growth in saline, 5-FU (25 mg/kg), and 4DPG (25 mg/kg) treated groups of animals. **C**, **D** Quantification of tumor volume and number of polyps per rat in above groups of animals. *n* = 6 rats per group, error bars: mean ± SD; *P < 0.05, **P < 0.01, ***P < 0.001. **E** Representative images of the lungs dissected from the above groups of animals showing metastatic tumor burden. Orange arrows indicating bumps of secondary tumors over each lobe of lungs. **F**, **G** Quantification of a number of macroscopic secondary tumors over the lung tissues and lung weight in the above groups of animals. *n* = 6 rats per group, error bars, mean ± SD; **P < 0.01, ***P < 0.001. **H** Immunohistochemistry analysis results showing protein expression of p-Chk2 (Thr68), Chk2, p53, p-Vimentin (Ser38), and Vimentin in tumor tissues isolated from saline and 4DPG (25 mg/kg) treated groups of animals. Scale bar: 50 µm. **I** Western blots depicting expression of p-Chk2 (Thr68), Chk2, p53, p-Vimentin (Ser38), and Vimentin in tumor tissues obtained from saline- and 4DPG-treated groups of animals. β-Actin expression was taken as endogenous loading control. **J** Densitometry analysis of the above western blot bands portraying relative protein expression of the markers presented. *n* = 3, error bars: mean ± SD; **P < 0.01, ***P < 0.001.

To examine the antitumor and anti-metastatic efficacy of 4DPG in vivo, we employed a carcinogen-induced orthotopic rat colorectal carcinoma model. The procedure is well established in our laboratory^41^, and yields substantially reproducible results. The schematic representation briefly describes the steps involved in the generation of orthotopic colorectal tumors along with the treatment strategy and further analysis (details in “Materials and methods”). The animals were euthanized at the end of the experiment and evaluated for tumor and metastatic burden (Fig. 4A). The control/saline-treated group of animals showed considerably larger tumors, along with numerous polyps covering the colorectal mucosa, with a mean tumor volume of 820.6 mm^3^. Conversely, the mean tumor volume was significantly reduced: 231 mm^3^ in the positive control (5-FU, 25 mg/kg) and 116.8 mm^3^ in 4DPG (25 mg/kg) treated groups of animals. The formation of colorectal polyps also condensed dramatically; an average number of polyps:16 in the control group of animals versus 8 in 5-FU and 3 in 4DPG-treated groups of animals (Fig. 4B–D). Further, lumps of secondary/metastatic tumors were clearly visible (orange arrows) over the lungs dissected from the control group of animals, whereas, 4DPG treatment strongly prevented the development of lung metastases. Although 5-FU treatment suppressed primary tumor growth; however, it could not restrict the metastatic spread of tumor cells to lung tissues. Abundant metastatic lumps were noticeable covering the lobes of lungs dissected from the 5-FU-treated groups of animals (Fig. 4E–G). Importantly, the groups of animals treated with 5-FU or 4DPG were healthy until the end of the experiment, without any drug-related toxicity/adverse effects. To validate whether our in vitro observations on mechanistic inhibition of EMT and invasion by 4DPG hold true in vivo, we performed immunohistochemical analysis of the tumor tissues acquired from the saline and 4DPG-treated animal groups. Surprisingly, a marked increase in protein levels of p-Chk2 (Thr68), and p53 concomitant with decreased p-Vimentin (ser38) and Vimentin expression levels were observed in tumor tissues extracted from the 4DPG-treated animal group compared to the saline-treated group (Fig. 4H). Finally, our western blotting analysis also corroborated substantial activation of p-Chk2 (Thr68) (3.54-fold) and p53 (5.82-fold) concurrent with downmodulation of p-Vimentin (ser38) (18.2-fold) and Vimentin (8.3-fold) in tumor tissues from 4DPG-treated group of animals compared to the saline treatment group (Fig. 4I, J). Taken together, the above-mentioned data strongly implies that 4DPG is a safe, well tolerable, and a potential inhibitor of colorectal tumor growth and metastases.

## Discussion

**Fig. 5:**
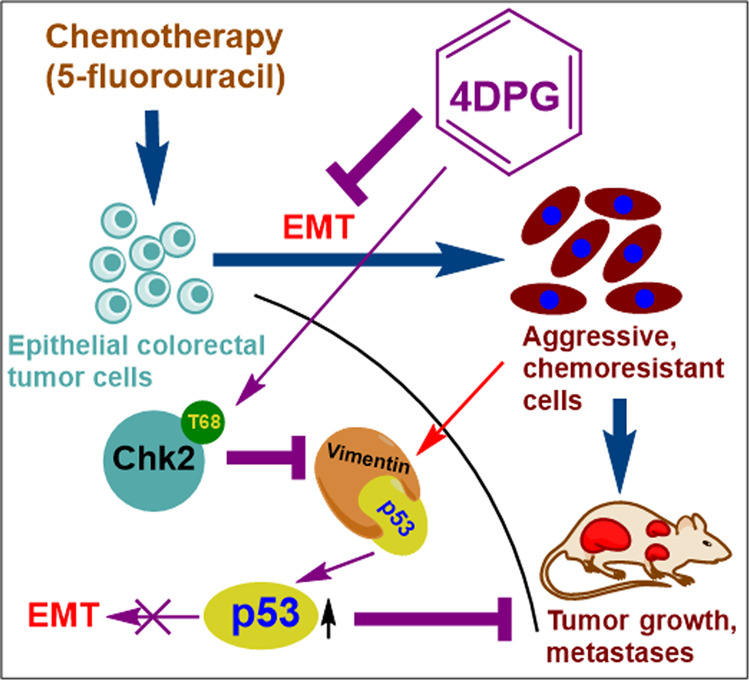
Schematic representation depicting the proposed mechanism of action of 4DPG. Epithelial CRC cells exposed to chemotherapeutic agents (5-FU) undergo EMT and transform into mesenchymal phenotypes. On the other hand, 4DPG treatment activates tumor-suppressor protein, Chk2, represses Vimentin, and disrupts Vimentin–p53 association in aggressive 5-FU-R CRC cells. This role of 4DPG further rescues/augments p53, suppresses EMT, and CRC progression.

In this study, we present evidence that CRC cells exposed to the chemotherapeutic drug (5-FU) (either transient exposure or 5-FU-R cells) gradually develop EMT phenotypes along with amplified Vimentin expression and invasive properties. We demonstrate that 4DPG, a natural product anticancer lead molecule activates Chk2 in a dose-dependent manner and suppresses the invasive capabilities of 5-FU-R cells. Moreover, 4DPG strongly downmodulates Vimentin phosphorylation and expression in a Chk2-dependent manner and promotes tumor suppressor protein, p53, to control these aggressive cells (Fig. 5). Together, our findings demonstrate the significance of Vimentin expression in conferring resistance to cancer cells as well as the efficiency of Chk2 in suppressing Vimentin and rescuing p53, ultimately circumventing drug resistance. The successful establishment of an EMT phenotype is distinguished by diminished expression levels of adhesion proteins, such as E-cadherin, and enhanced expression levels of the mesenchymal proteins, like fibronectin and Vimentin^14^. Clinically, many cancers display reduced E-cadherin and increased Vimentin expression that aids tumor progression and metastasis^14,42^.

Vimentin is an intermediate filament protein that upholds cellular structure and integrity in normal mesenchymal cells. Importantly, during metastasis Vimentin regulates the cell shape and migration ability to support the EMT phenomenon^35,36,43^. A study demonstrated decreased migration and invasion upon Vimentin knockdown in CRC cells, underlining the significance of Vimentin in regulating EMT^44,45^. In addition, Vimentin overexpression was reported to be linked to enhanced metastatic ability and chemoresistance in several cancer types^46,47^. Colonic neoplastic (SW-620) cells highly express Vimentin, which is correlated with EMT, resistance to histone deacetylase inhibitors, and disease progression^45^. Further, expression of Vimentin in the stroma of colorectal tumors reflect higher malignant potential, poor survival, and disease recurrence in patients with CRC^48^. In this regard, our recently reported research work has demonstrated activation of Vimentin during early apoptosis, which confers a survival advantage to CRC cells treated with DNA-damaging anticancer agents^49^. Indeed, Vimentin plays a crucial role in nurturing the metastatic cancer cells and aiding their escape from the cytotoxic effects of chemotherapeutic agents, thus fostering drug resistance.

Tumor suppressor p53 serves as a guardian of the genome that controls hyperactivation of numerous oncogenes through various mechanisms. Also, the expression of p53 is considered as a prognostic marker that greatly determines the therapeutic outcome in neoplastic diseases. However, mutations in p53 are frequently observed in many human malignancies including breast, colorectal, and glioblastomas, which later paves the way for cancer progression^50,51^. Pro-EMT genes and transcription factors are also increasingly recognized as repressors of p53 functions; For example, Twist1, a mesenchymal marker and transcription factor, is involved in the degradation of p53 in sarcomas. A study by Piccinin et al. demonstrated the existence of a Twist-box code at the C-terminus of p53 to which Twist1 binds and facilitates the inactivation of p53 by murine double minute (MDM2)^52^. In rheumatoid arthritis synovial fibroblasts (RASFs), Vimentin forms complex with p53 in the cytosol, which prevents the nuclear translocation of p53 and hinders its subsequent functions. In this study set-up, caspase-4-cleaved Vimentin and released cytosolic p53 leading to its nuclear translocation. This role of caspase-4 further induced apoptosis in RASFs via the tumor necrosis factor-related apoptosis-inducing ligand (TRAIL-R2). Conversely, blockade of caspase-4 with specific SiRNAs or small-molecule inhibitor showed absence of p53 nuclear translocation and restrained TRAIL-R2-mediated apoptosis^53^. From that perspective, our data for the first-time unveiled association between Vimentin and p53 in 5-FU-R-aggressive CRC that plausibly accounts for the reduced nuclear expression of p53 and its consequent functions. On the other hand, 4DPG treatment effectively dissociated this interaction promoting the nuclear expression of p53 (Fig. 3). These data not only help establish their association and functional role as biomarkers of metastasis but also reveal the clinical significance of therapeutic targeting of such molecular complexes for limiting cancer progression and chemoresistance.

We previously reported that modulation of Chk2 via genetic or pharmacological methods (treatment with 4DPG) attenuated Twist1-mediated EMT and invasion in p53-defective cancer cells. Chk2 activation also repressed Vimentin expression along with ZEB-1 and MMP-2, while augmenting epithelial markers E-cadherin and TIMP-1^31^. Since Vimentin is a downstream target gene of Twist1 during EMT and both these markers cooperatively facilitate the invasion–metastasis cascade^54^, it seems rational that Chk2 can curtail Vimentin activation by regulating Twist1. Intriguingly, pharmacological activation of Chk2 (Thr68) by 4DPG efficiently downregulated Twist1 along with p-Vimentin (Ser38) and Vimentin expression; thus, reducing the invasion abilities of 5-FU-R cells. Moreover, 4DPG potentially inhibited colorectal tumor growth, polyps formation, and prevented lung metastases burden compared to the standard chemotherapeutic agent, 5-FU, at a similar dose. In addition, 4DPG is hemocompatible, well tolerable by the animals, and possesses suitable pharmacokinetic properties, which warrants its therapeutic development for the treatment of metastatic diseases.

## Materials and methods

### Cell culture

Cell lines used in this study were procured from the American Type Culture Collection (Manassas, VA). HCT-116 and SW-620 cells were cultured in RPMI-1640 medium and HT-29 cells in McCoy’s 5a medium supplemented with 10% fetal bovine serum (Gibco, Carlsbad, CA) and 1% penicillin/streptomycin (Sigma-Aldrich, St. Louis, MO) in a humidified CO_2_ incubator (New Brunswick, Galaxy 170R) at 37 °C and 5% CO_2_. Cells were passaged regularly after 2–3 days to maintain a healthy growth rate and periodically tested for mycoplasma contamination.

### Antibodies, chemicals, and reagents

Primary antibodies such as anti-Vimentin (#sc-5565), anti-p-Vimentin (Ser38) (#sc-16673), anti-Chk2 (#sc-9064), anti-Twist1 (#sc-15393), anti-MMP-2 (#sc-10736), anti-E-cadherin (#sc-7870), anti-TIMP-1 (#sc-5538), and anti-p53 (#sc-393031) were obtained from Santa Cruz Biotechnology (Santa Cruz, CA). Anti-p-Chk2 (Thr68) (#2661) was procured from Cell Signaling Technologies (Beverly, MA). Alexa Fluor (488)-conjugated secondary anti-rabbit antibodies (#A-11094) was purchased from Thermo-Fisher Scientific (Waltham, MA). Anti-β-actin (#A2228), horseradish peroxidase (HRP)-conjugated goat anti-rabbit IgG (#A6154), anti-mouse IgG (#A3673), and all chemicals/reagents including dimethylsulfoxide (DMSO), 4′,6-diamidino-2-phenylindole (DAPI),5-fluorouracil, crystal violet, fluorescein-5-isothiocyanate (FITC), and Chk2-inhibitor (PV1019) were purchased from and Sigma-Aldrich (St. Louis, MO).

### Generation of 5-FU-resistant (5-FU-R) cell lines

Previously described procedure by Shukla et al. was followed with minor modifications^55^. In brief, wild-type CRC cells (HCT-116, SW-620, and HT-29) were cultured with increasing concentrations of 5-FU for at least 20 weeks. The acquired drug resistance was tested periodically by determining the half maximal inhibitory concentration (IC_50_) of 5-FU through MTT cytotoxicity assays. The technique generated aggressive, drug-resistant HCT-116/5-FU-R, SW-620/5-FU-R, and HT-29/5-FU-R cells.

### MTT cytotoxicity assay, Matrigel invasion assay, and fluorescent gelatin degradation assay

The experiments were performed using the wild-type and 5-FU-RCRC cells following our standardized protocols, as described previously^56,57^.

### Transient transfection

HCT-16/5-FU-R and SW-620/5-FU-R cells were plated over the coverslips (4 × 10^5^) in six-well plates (for immunocytochemistry) or seeded into the upper chambers of invasion assay system (5 × 10^4^) and followed by transfection. Dr. Domenico Delia (Fondazione IRCCS Istituto Nazionale Tumori, Italy) generously gifted the GFP, GFP-Chk2, pCDNA3, and/or HA-Chk2 plasmid constructs that were transfected to the cells using Lipofectamine-3000 reagent (Thermo-Fisher Scientific, Carlsbad, CA) following the instructions by the manufacturer.

### SiRNA knockdown experiments

Sigma-Aldrich (St. Louis, MO) provided the MISSION eSiRNAs against human *CHEK2* (EHU158481). Cells were seeded over the coverslips or into the upper inserts of the Boyden chamber and transfected using Lipofectamine-3000 reagent (Life Technologies, Carlsbad, CA) following the instructions by the manufacturer.

### Western blotting

We adhered to the previously published protocol for western blotting^56^. To sum up, cells were exposed to the respective treatments (mentioned in figure legends). Post-treatment, cells were lysed with cell lysis buffer, and cell lysates were collected after centrifugation. Bradford assay was performed to quantify the protein concentration. Equal amount of protein was taken from each sample to carry out the western blot analysis. SDS-PAGE was employed to separate the proteins that were later transferred onto PVDF membranes (Millipore, Billerica, MA). Next, the membranes were blocked with 5% skim milk and subsequently incubated with the specific primary antibodies (1:1000 dilution), either for 3 h at room temperature or overnight at 4 °C. Later, the blots were washed thoroughly with TBST and probed with the respective secondary antibodies (1:2000 dilution) coupled with horseradish peroxidase for 1 h. Following thorough washing, the membranes were visualized using enhanced chemiluminescence reagent (Millipore) and captured onto Biomax light film (The Eastman Kodak Co., Rochester, NY).

### Immunocytochemistry

We followed the standard protocol with some modifications for immunocytochemistry^56,58^. In brief, cells, at a density of 4 × 10^5^ cells/well, were seeded over the sterile coverslips placed in six-well plates. Following the respective treatment, cells were washed thrice with PBS. Next, the cells were fixed with 4% paraformaldehyde (pH = 7.4) for 15 min, permeabilized with 0.3% Triton X-100 for 10 min, and then blocked with 1% goat serum for 30 min. Consequently, the cells were incubated with anti-Vimentin antibody overnight at 4 °C, at a dilution of 1:100, followed by washing with PBST and further incubation with secondary anti-rabbit Alexa Fluor 488 (1:750 dilution). After subsequent washing and mounting, the images were captured at ×20 magnification under Floid Cell Imaging Station (Invitrogen).

### Immunoprecipitation analysis

For immunoprecipitation analysis, the previously described procedure was followed with minor modification^31^. Briefly, SW-620/5-FU-R cells (2 × 10^6^) were seeded in 90-mm Petri dishes and incubated in a humidified CO_2_ incubator for 24 h. After preparation of cell lysates and subsequent preclearing with protein G PLUS-Agarose beads, immunoprecipitation was carried out with the precleared lysates using 5 μg of p53 or Vimentin antibody conjugated to 50 μl of protein G PLUS-Agarose beads. Following careful washing with lysis buffer, the immunoprecipitates were analyzed by western blotting.

### Cellular fractionation studies

SW-620/5-FU-R cells seeded in 90-mm Petri dishes were treated with vehicle and/or 4DPG for 48 h, following overnight incubation. Cells were then harvested, washed with PBS; cytosolic and nuclear extracts were prepared following our established protocol^37,56^.

### Distribution coefficient, solubility, and hemolysis determination

The distribution coefficient, solubility, and hemocompatibility of 4DPG was determined adopting our previously described protocol^37,40^. For hemolysis assay, increasing concentrations (5, 10, and 20 µg/mL) of 4DPG were employed. Distilled water (DW) and PBS were considered as negative and positive control, respectively.

### Pharmacokinetic (PK) study

The PK studies were performed following our standardized procedure with minor modifications^37^. To determine the PK profile of 4DPG, healthy male Balb/c mice (body weight 25–30 g each) were employed in the study. Six animals were enrolled for each sampling time point and a total of 11 time points (0.083, 0.25, 0.5, 1, 2, 4, 6, 8, 12, 16, and 24 h) were involved in the study. Control group of mice were administered with saline and test groups of animals received a single dose of 4DPG (5 mg/kg, body weight) intravenously. After the successful injections of the molecule, 100 µL of plasma was collected from each animal enrolled at the above sampling time points and the plasma proteins were precipitated with 400 µL of acetonitrile. Following extraction into the solvent and subsequent filtration, the compound was analyzed using a 6410 triple-quadrupole LC–MS/MS system (Agilent Technologies, USA). Individual samples collected at each time point were quantified through single ion monitoring (SIM) by comparing with the standard calibration curve prepared using Agilent Mass Hunter software (version B.04.00) (detailed description is provided in Supplementary information file). Essential PK parameters such as half-life (*t*_1/2_), C initial (C_0_), area under the curve (AUC_0–∞_), volume of distribution (Vd), clearance (Cl), and mean residence time (MRT) were calculated utilizing noncompartmental pharmacokinetic data analysis.

### Animal model for colorectal carcinogenesis

Animal studies were performed following the guidelines approved by Institutional Animal Ethics Committee (IAEC No. 51/02/15) of CSIR-Indian Institute of Integrative Medicine, Jammu, India. The experiment was performed according to our standardized protocol described previously with necessary modifications^41^. Five-weeks-old healthy male Wistar rats (body weight 150–200 g) were maintained in aseptic condition with free access to food and water. Animals were randomized into three groups, with six animals per group. All the rats received intrarectal instillation of 0.5 mL of freshly prepared 0.4% aqueous solution of N-methyl-N-nitrosourea (MNU) thrice a week for 3 weeks, for the orthotopic induction of colorectal tumors. The solution was injected using sterile syringe with attached 8-cm long rubber catheter tube inserted two-thirds into the colon lumen through anal orifice, as described previously^59^. The animals were kept under continuous monitoring, and the body weight was measured once per week. Following 22 weeks after the initiation of MNU administration, treatment regimens were started by administering 5-FU (25 mg/kg), and 4DPG (25 mg/kg) thrice a week for 3 weeks. The control and treatment groups of animals were dosed separately by three independent investigators and the outcomes were assessed by a fourth investigator blind about the dosing process and treatment regimen. Animals were euthanized at the end of 25 weeks, the large bowel and lungs were dissected and thoroughly evaluated for the presence of polyps/tumors/metastatic nodules. Relevant tissues were preserved for further biochemical analysis.

### Immunohistochemical analysis

The procedure was performed following our pre-standardized protocol with minor modifications^31^. Paraffin-embedded tissue sections were prepared from the tumors of saline, 5-FU, and 4DPG-treated groups of animals. Primary antibodies used: p-Chk2 (T68), Chk2, p53, p-Vimentin (S38) and Vimentin at 1:150 dilution for overnight at 4 °C.

### Statistical analysis

Data were expressed as the mean ± SD of at least two independent experiments performed. Analysis was performed using either Student’s *t* test or one-way ANOVA according to the experimental conditions. A two-sided value of *P < 0.05 was considered significant in all cases.

## Supplementary information

Supplementary Figure S1

Supplementary Figure S2

Supplementary Figure S3

Supplementary Figure S4

Supplementary methods

Supplementary Figure Legends
