# Supplementary figures and images for: Natural podophyllotoxin analog 4DPG attenuates EMT and colorectal cancer progression via activation of checkpoint kinase 2
Source: Cell Death Discov. 2021 Jan 26;7:25. doi: 10.1038/s41420-021-00405-3 (PMC7838189; doi:10.1038/s41420-021-00405-3)

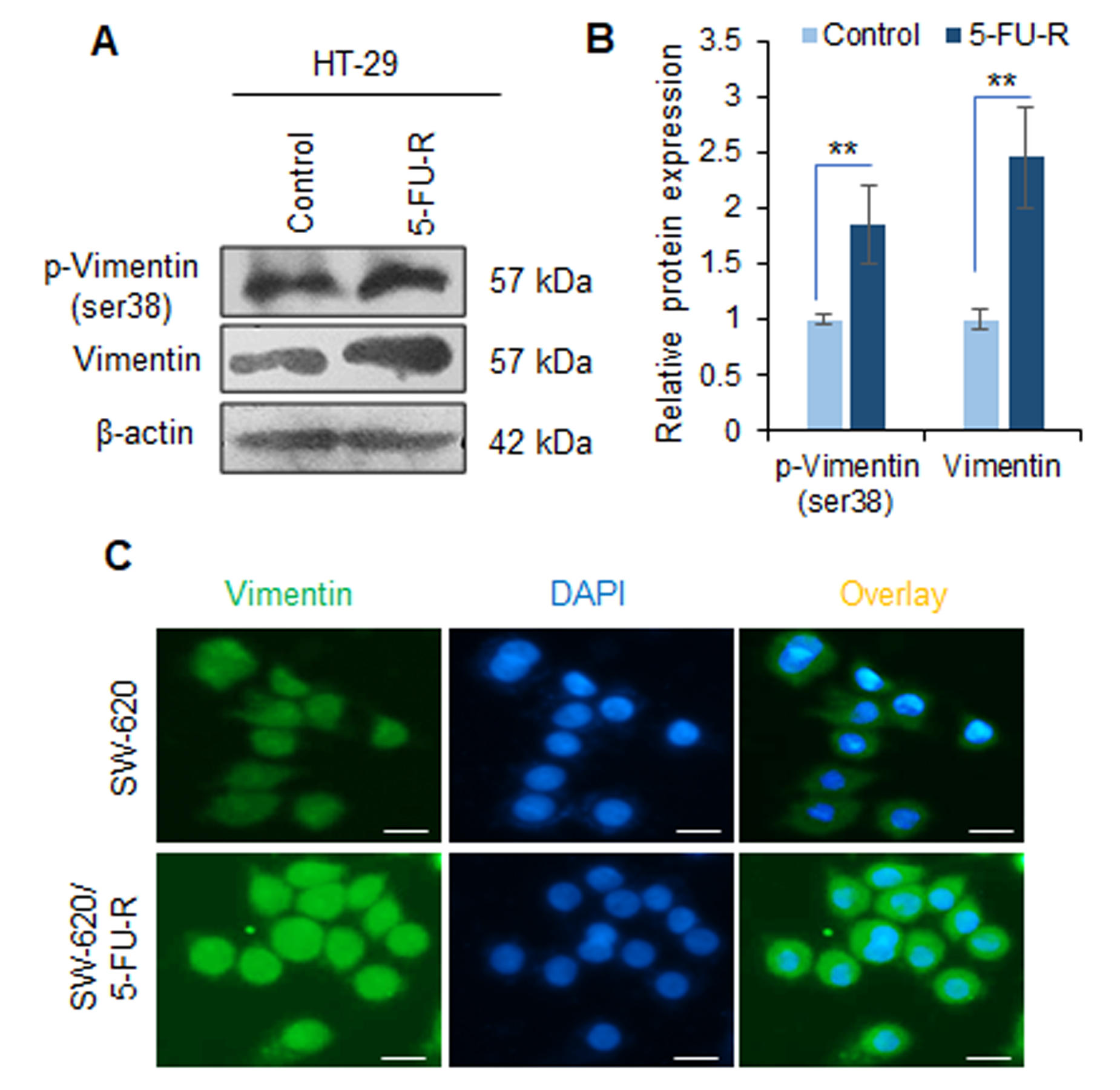

Supplement: Supplementary file 1 — Supplementary Figure S1 [file 41420_2021_405_MOESM1_ESM.tif]

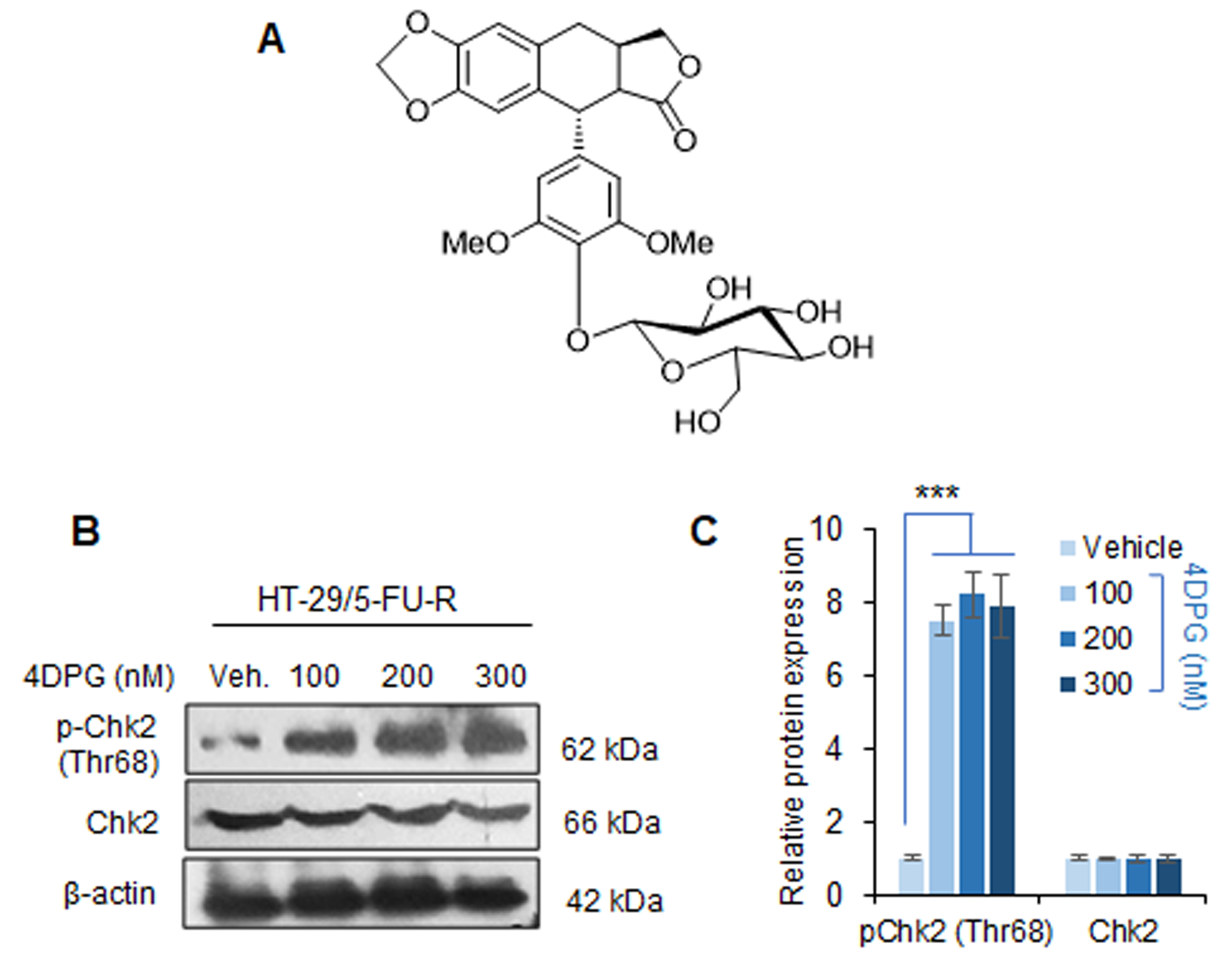

Supplement: Supplementary file 2 — Supplementary Figure S2 [file 41420_2021_405_MOESM2_ESM.tif]

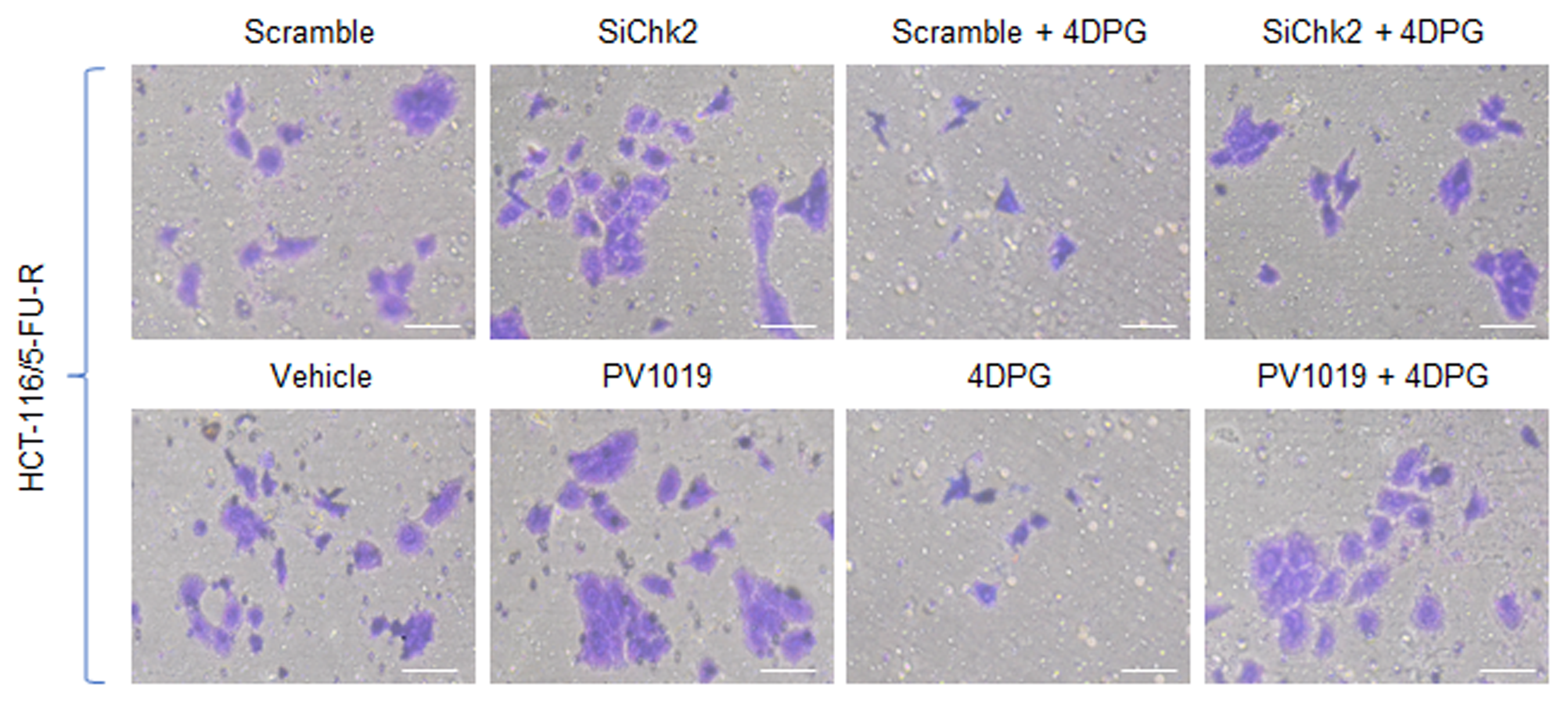

Supplement: Supplementary file 3 — Supplementary Figure S3 [file 41420_2021_405_MOESM3_ESM.tif]

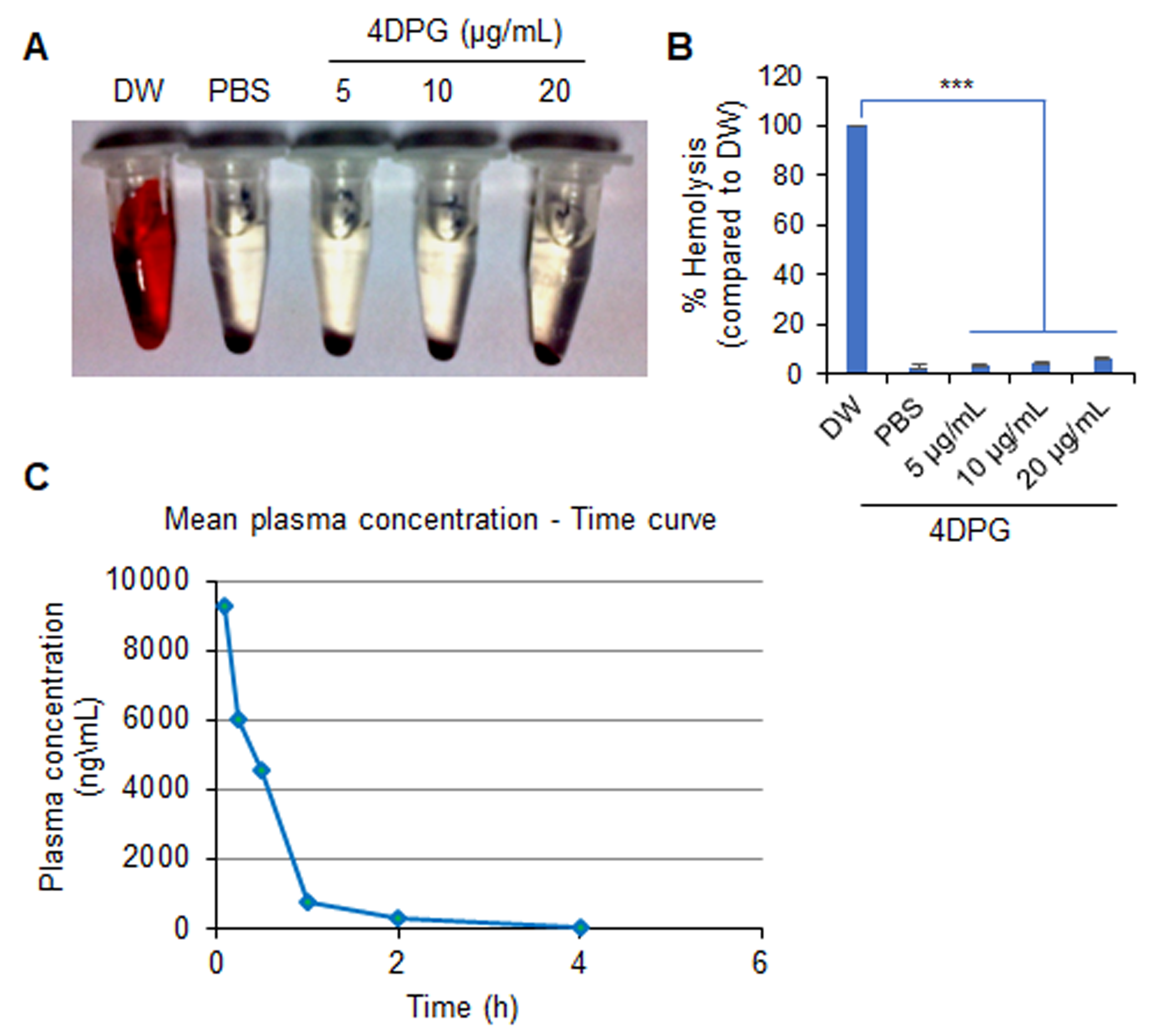

Supplement: Supplementary file 4 — Supplementary Figure S4 [file 41420_2021_405_MOESM4_ESM.tif]
