# Supplementary material for: Natural podophyllotoxin analog 4DPG attenuates EMT and colorectal cancer progression via activation of checkpoint kinase 2
Source: Cell Death Discov. 2021 Jan 26;7:25. doi: 10.1038/s41420-021-00405-3 (PMC7838189; doi:10.1038/s41420-021-00405-3)
Supplement: Supplementary file 5 — Supplementary methods [file 41420_2021_405_MOESM5_ESM.docx]

**Natural podophyllotoxin analog 4DPG attenuates EMT and colorectal cancer progression via activation of checkpoint kinase 2**

**Supplementary methods:**

**Details of LC-MS/MS analysis performed for the pharmacokinetic study**

**Chemicals and reagents**

MS-grade Acetonitrile, water and formic acid were purchased from Merck (Merck,Darmstadt, Germany).

**Stock solution of standard compound**

Stock solution of 4DPG (1mg/mL) was prepared in volumetric in the mobile phase. Standard working solutions were then obtained by mixing and making appropriate dilutions of stock solutions using mobile phase. The concentration utilized for the preparation of seven-point calibration curve ranged between 0.01 to 10,000 ng/mL. Standard solution was filtered through a 0.2 µm membrane filter (Millipore) and injected directly. The stock and working solution were stored at +4 °C.

**Calibration curve**

Before making calibration curve one blank injection was run to check the noise level of the system. The calibration equation of 4DPGwas obtained by plotting LC-MS peak area (y) versus the concentration (x, ng/mL) of calibrators as y= 75.536575x+836.414260 (r^2^=0.9971). The equation showed very good linearity over the range.

**HPLC-ESI-MS/MS analysis**

Experiments were performed on an Agilent 1260 Infinity (Agilent, USA) HPLC system coupled with an Agilent 6410 (Agilent Technologies, USA) triple quadrupole MS/MS instrument equipped with an ESI ion source that was used in the positive ion mode. Chromatographic separation was performed on a Chromolith high resolution RP18e column (100 x 4.6 mm) maintained at 30^0^C. Mobile phases of 0.1% (*v/v*) formic acid in water (eluent A) and acetonitrile (eluent B) were used at a flow rate of 350 µL/min and injection volume was 10µL. The isocratic elution was taken as programmed at A with 20% and B with 80%. Data handling was performed using Mass Hunter workstation. The following MS parameters were applied to the method using single ion monitoring (SIM): capillary voltage 4000 dwell time of 50 ms and a step size of 0.1 amu. Data were acquired in centroid mode from 100–600 mass-to-charge ratio (m/z) in MS scanning. To get maximum resolution spectrometric conditions, fragmentor voltage of the investigated compound was optimized. Maximum resolution for the 4DPG was obtained at fragmentor voltage of 270V. The sodium adduct species [M+Na]^+^of 4DPG (m/z 569.1), was selected as precursor ions.Compound was identified by comparison of sodium adduct ion, and retention time with that of the standard compound. The developed method for 4DPG showed retention time of 4.46 min.
