# Supplementary material for: Natural podophyllotoxin analog 4DPG attenuates EMT and colorectal cancer progression via activation of checkpoint kinase 2
Source: Cell Death Discov. 2021 Jan 26;7:25. doi: 10.1038/s41420-021-00405-3 (PMC7838189; doi:10.1038/s41420-021-00405-3)
Supplement: Supplementary file 6 — Supplementary Figure Legends [file 41420_2021_405_MOESM6_ESM.doc]

**Natural podophyllotoxin analog 4DPG attenuates EMT and colorectal cancer progression via activation of checkpoint kinase 2**

**Supplementary Figure Legends:**

**Supplementary Figure S1.** 5-FU exposure augments Vimentin expression in CRC cells. **(A)** Western blots depicting protein expression of pVimentin (Ser38) and Vimentin in wild-type (control) versus HT-29/5-FU-R cells. Beta actin expression was considered as endogenous loading control. **(B)** Densitometry analysis showing the relative protein expression in each band of western blots presented above. n=3, error bars: mean ± SD; **P<0.01. **(C)** Immunocytochemistry results displaying Vimentin expression level in wild-type (SW-620) versus SW-620/5-FU-R cells. Scale bars: 50 µm.

**Supplementary Figure S2.** 4DPG induces Chk2 in 5-FU-R CRC cells. **(A)** Structure of 4DPG (4′-demethyl-deoxypodophyllotoxin glucoside). **(B)** Western blotting analysis results portraying protein expression of pChk2 (Thr68) and Chk2 in HT-29/5-FU-R cells in response to increasing concentrations of 4DPG treated for 48 h. Beta actin expression was considered as endogenous loading control. **(C)** Densitometry analysis showing relative protein expression of western blot bands presented above. n=3, error bars: mean ± SD; ***P<0.001.

**Supplementary Figure S3.** 4DPG inhibits invasion of 5-FU-R cells in a Chk2-dependent manner. Matrigel invasion assay results showing the representative images of invaded HCT-116/5-FU-R cells after 48 h of treatment/transfection with scramble, SiChk2, scramble plus 4DPG, SiChk2 plus 4DPG, vehicle, PV1019, 4DPG, and PV1019 plus 4DPG. Scale bars: 50 µm.

**Supplementary Figure S4.** Hemocompatibility and exposure of 4DPG. **(A)** Representative images of RBC suspension incubated with distilled water (negative control), PBS (positive control), and increasing concentrations of 4DPG for 1 h. **(B)** Quantification of hemolysis in above treatment conditions by measuring the optical density at 540 nm. Error bars: mean ± SD; ***P<0.001. **(C)** Mean plasma concentration versus time curve obtained from the pharmacokinetic analysis showing plasma concentration of 4DPG over time in Balb/c mice administered intravenously with single dose (5 mg/kg/body weight) of the compound.
